# Bioactive Potential and Chemical Composition of *Vitex agnus-castus* L. Leaf Extracts Collected in Algeria: A Combined In Vitro and In Silico Approach

**DOI:** 10.3390/molecules30030749

**Published:** 2025-02-06

**Authors:** Amina Bramki, Ghozlane Barboucha, Ouided Benslama, Anna Andolfi, Fatima Zohra Makhlouf, Maria Smati, Djamila Benouchenne, Mohamed Moussaoui, Chawki Bensouici, Alessio Cimmino, Jesús G. Zorrilla, Maria Michela Salvatore, Marco Masi

**Affiliations:** 1 Higher National School of Biotechnology Taoufik KHAZNADAR, Nouveau Pôle Universitaire Ali Mendjeli, BP. E66, Constantine 25100, Algeria; a.bramki@ensbiotech.edu.dz (A.B.); g.barb@ensbiotech.edu.dz (G.B.); makhlouf_f.zohra@umc.edu.dz (F.Z.M.); m.smati@ensbiotech.edu.dz (M.S.); d.benouchenne@ensbiotech.edu.dz (D.B.); 2 Laboratory of Natural Substances, Biomolecules, and Biotechnological Applications, Department of Natural and Life Sciences, Larbi Ben M’Hidi University, Oum El Bouaghi 04000, Algeria; ouided.benslama@univ-oeb.dz; 3 Department of Chemical Sciences, University of Naples Federico II, 80126 Naples, Italy; andolfi@unina.it (A.A.); alessio.cimmino@unina.it (A.C.); jesus.zorrilla@uca.es (J.G.Z.); 4 Pharmaceutical Sciences Research Center, Constantine 25100, Algeria; mohamed.moussaoui@univ-bejaia.dz; 5 Biotechnology Research Center, UV 03, BP. E73, Ali Mendjeli, Constantine 25016, Algeria; c.bensouici@crbt.dz; 6 Allelopathy Group, Department of Organic Chemistry, Facultad de Ciencias, Institute of Biomolecules (IN-BIO), University of Cadiz, 11510 Puerto Real, Spain; 7 Department of Veterinary Medicine and Animal Production, University of Naples Federico II, 80137 Naples, Italy; mariamichela.salvatore@unina.it

**Keywords:** *Vitex agnus-castus*, chemical composition, antioxidant assays, enzymatic assays, antibacterial assays, molecular docking

## Abstract

*Vitex agnus-castus* L., a medicinal plant widespread in the Middle East and Europe, is traditionally used to treat various disorders. In this study, extracts from its leaves, collected in Algeria, were evaluated for their antioxidant, enzymatic, and antibacterial activities through in vitro and in silico studies. The hydroalcoholic extract was fractionated using solvents of varying polarity to isolate bioactive compounds with potential biological effects. Notable levels of total phenolics, flavonoids, and flavonols were detected in the dichloromethane (CH_2_Cl_2_) and ethyl acetate (EtOAc) extracts. NMR and GC-MS were used to identify metabolites in the extracts, which were discussed in relation to their biological activities. Antioxidant assays showed that the EtOAc extract had a remarkable effect, particularly in the DPPH^•^ free radicals test (IC_50_ = 15.68 ± 1.51 μg/mL), while enzymatic assays revealed that the dichloromethane extract moderately inhibited butyrylcholinesterase (IC_50_ = 133.54 ± 1.45 μg/mL). Antibacterial assays showed that the extracts inhibited the growth of *Staphylococcus aureus*, *Bacillus subtilis*, and *Escherichia coli* strains, with the most significant effect observed for the *n*-hexane extract, especially against *S. aureus* and *B. subtilis* (respectively, 22.33 ± 0.47 and 18.33 ± 0.47 mm diameters). These outcomes were validated via molecular docking simulations on three DNA gyrase enzymes: 3G7E (from *E. coli*), 3G75 (from *S. aureus*), and 4DDQ (from *B. subtilis*), revealing that linolenic and palmitic acids, as well as phytol significantly interacted with these enzymes, showing varying binding affinities and suggesting antibacterial potential against the targeted species *E. coli* and *S. aureus*. These findings highlight the potential therapeutic use of *V. agnus-castus* leaves, encouraging further research into their applicability in the development of plant-derived drugs.

## 1. Introduction

Over the past ten years, the use of alternative medicine has experienced significant global growth. This practice is no longer limited to developing countries but has also extended to nations where conventional medicine dominates healthcare systems. The prolonged use of plant-based medicines is an effective and economical solution for maintaining health while minimizing side effects. Today, phytotherapy is gaining increasing interest as a natural source of compounds with biological activities [1]. The genus *Vitex*, also known as chaste tree, is the largest in the Verbenaceae family, comprising around 270 species distributed worldwide [2]. Most *Vitex* species are deciduous shrubs or small trees [3]. They are primarily found in the temperate regions of Asia and the warm areas of Europe, with a strong presence in Southeast Asia. These species are widely used in traditional medicine, particularly in South Asian countries such as India, China, Nepal, Sri Lanka, Bangladesh, and Malaysia, but also in regions like Indonesia, Egypt, Iran, Morocco, Brazil, and Mexico. Recognized as valuable sources of medicine, *Vitex* species have been the subject of numerous studies [4]. Among them, *Vitex agnus-castus* L., widely distributed in the Middle East and Europe [5], has traditionally been used to treat various disorders such as gynecological issues, gastrointestinal problems, headaches, the flu, insufficient lactation, and acne [6,7]. Recent research has highlighted its effectiveness as a promising alternative in the treatment of premenstrual syndrome and postmenopausal syndrome, and in increasing insufficient milk production [7]. It is also recognized as one of the most effective medicinal plants for relieving uterine cramps [8]. Furthermore, it has been shown to possess various pharmacological properties, including antibacterial, antihistamine, anti-inflammatory, and antioxidant activities [1]. The study of the chemical composition, as well as the biological activities, of this species represents an integrative approach offering new opportunities for the design of novel natural therapeutic agents. In this context, this work aims to study the phytochemical profile of *V. agnus-castus* leaf extracts from Algeria using NMR and GC-MS analysis. Furthermore, the biological activities of these extracts were evaluated through in vitro and in silico approaches, with a focus on their antioxidant, acetyl- and butyrylcholinesterase inhibitory and antibacterial potentials.

## 2. Results and Discussion

### 2.1. Determination of Total Phenolic, Flavonoid, and Flavonol Contents (TPC, TFC, and TFolC)

Leaves from *V. agnus-castus* were extracted, as detailed in Materials and Methods, to obtain three different extracts, each based on the solvent used for extraction: *n*-hexane, dichloromethane, and ethyl acetate (EtOAc). The TPC, TFC, and TFolC in the three extracts are presented in Table 1.

The results revealed that the levels of TPC, TFC, and TFolC differ according to the extraction solvent used. Among the extracts, the EtOAc demonstrated the highest concentrations of TPC (203.00 ± 1.86 µg GAE/mg of extract), TFC (47.36 ± 0.76 µg QE/mg of extract) and TFolC (37.20 ± 1.80 µg QE/mg of extract), followed by dichloromethane, while in the *n*-hexane extract, only a moderate TFolC was detected (19.56 ± 0.51 µg QE/mg of extract). These results are consistent with those reported by Kimna and Fafal [9], who worked on ethanolic extracts of *V. agnus-castus* leaves and found phenolic compounds concentrations of 190 μg GAE/mg extract and flavonoid concentrations of 150 μg QE/mg extract. Moreover, our results surpass those reported by Berrani et al. [10], who reported phenolic compound concentrations of 5.50 ± 0.35 μg GAE/mg extract and flavonoid concentrations of 1.39 ± 0.17 μg QE/mg extract. The quantitative differences observed in flavonoids levels could be attributed to variations in extraction methods, geographical location, and climatic or environmental conditions [11,12]. To our knowledge, no studies have been conducted to quantify the concentration of flavonols in the leaves of *V. agnus-castus* species. Moreover, our results showed that the TPC, TFC, and TFolC detected in extracts obtained using solvents with lower polarity, such as *n*-hexane and dichloromethane, were relatively low compared to those extracted using EtOAc, a more polar solvent. This observation aligns with the fact that polar solvents, such as EtOAc, methanol, and water, tend to extract higher concentrations of phenolic compounds and flavonoids, as a consequence of strong hydrogen bonding and dipole–dipole interactions between the polar solvent molecules and the hydroxyl groups of these phenolic compounds, which enhance their solubility in polar media [13]. In contrast, less-polar solvents like *n*-hexane exhibit a preferential affinity for nonpolar or low-polarity phenolic compounds, primarily due to the inability of the solvent to disrupt intramolecular hydrogen bonds in more hydrophilic compounds, and the extraction efficiency of polar compounds is markedly reduced in these solvents [14].

### 2.2. Chemical Characterization

Organic extracts from *V. agnus-castus* leaves, obtained as reported in Material and Methods, were analyzed via NMR and GC-MS. ^1^H NMR spectra of the three organic extracts (Figures S1–S3) showed signals belonging to different classes of natural products [15]. In particular, the ^1^H NMR spectrum of the *n*-hexane extract (Figure S1) showed in the region of olefinic protons a double doublet at δ 5.84 (*J* = 10.1, 17.6 Hz) coupled with two broad doublets at δ 5.18 (*J* = 17.6 Hz) and 5.05 (*J* = 10.1 Hz), probably due to the three vinylic protons of an ABX spin system [16], and a signal at δ 5.46–5.33 typical of protons of unsaturated fatty acid double bonds [17]. Furthermore, the same spectrum showed as other main signals the singlets probably attributed to methyl protons on sp2 carbons at δ 2.06–1.97 and the signals of aliphatic methylene and methyl protons [18]. The ^1^H NMR spectrum of the dichloromethane (CH_2_Cl_2_) extract (Figure S2) showed typical signals of aromatic protons at δ 8.17–6.81 and protons of methoxy groups resonating as singlets at δ 4.02–3.90, suggesting the presence of substituted benzene derivatives [18]. Finally, the ^1^H NMR spectrum of the EtOAc extract (Figure S3) showed, as main signals, the doublets of *p*-disubstituted aromatic rings at δ 7.95–7.89 and at δ 6.87–6.82 [18].

**Figure 1:**
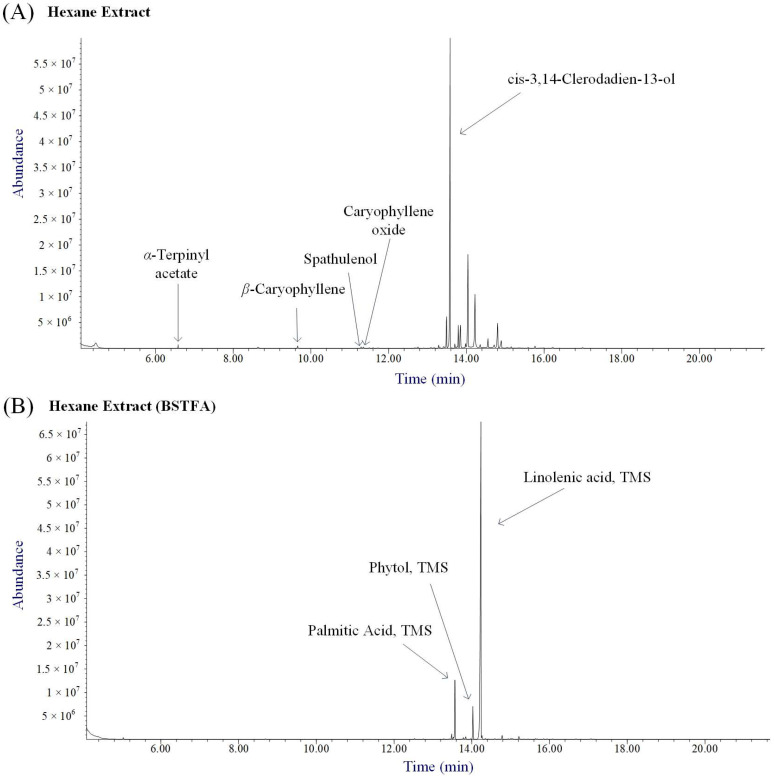
Annoted total ion chromatograms (TICs) acquired (**A**) directly and (**B**) after derivatization with *N*,*O*-bis(trimethylsilyl)trifluoroacetamide (BSTFA) of the *n*-hexane extract of *V. agnus-castus*.

Based on the NMR spectrum, the compounds present in the *n*-hexane extract may exhibit a range of polarities and physicochemical properties. For this reason, the sample was analyzed both directly via GC-MS, and after derivatization with *N*,*O*-bis(trimethylsilyl)trifluoroacetamide (BSTFA) to enhance volatility and improve detection of specific analytes. As shown in Figure 1A, the volatile fraction of the *n*-hexane extract includes five compounds belonging to the terpenoid class that have been previously reported as components of aerial part extracts of *V. agnus-castus* [19,20]. Figure 1B presents the total ion chromatogram of the *n*-hexane extract, which was trimethylsilylated prior to injection into GC-MS. Two fatty acids (i.e., palmitic and linolenic acids) were identified along with phytol.

**Figure 2:**
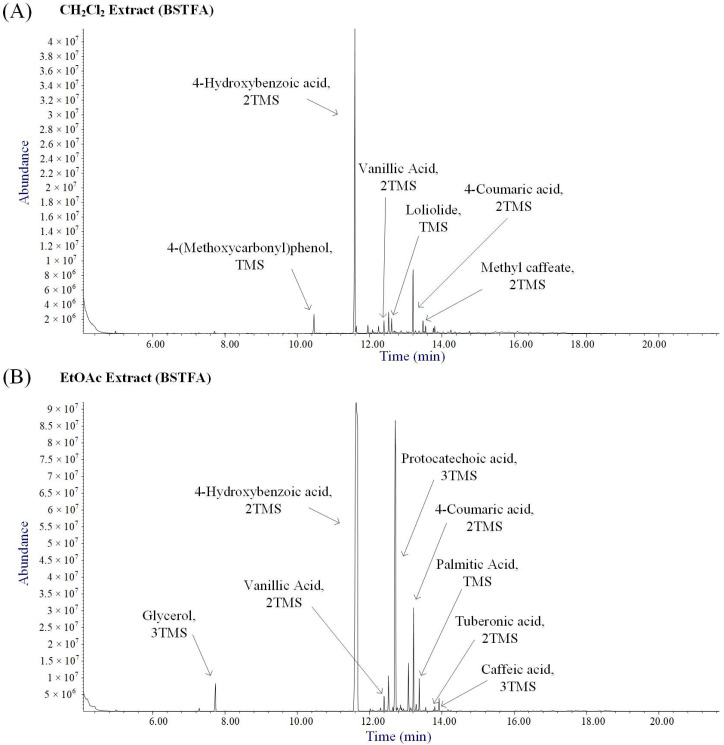
Annoted total ion chromatograms (TICs) acquired after derivatization with *N*,*O*-bis(trimethylsilyl)trifluoroacetamide (BSTFA) of the (**A**) CH_2_Cl_2_ and (**B**) EtOAc extracts of *V. agnus-castus*.

The GC-MS analysis of the CH_2_Cl_2_ (Figure 2A) and EtOAc (Figure 2B) extracts, conducted after derivatization with BSTFA, revealed the presence of several phenolic compounds, including 4-hydroxybenzoic acid, protocatechuic acid, vanillic acid, and caffeic acid. These findings confirm that the leaves of *V. agnus-castus* are a rich source of phenolic compounds and their derivatives, which may exhibit a broad spectrum of biological activities [21,22].

### 2.3. Antioxidant Activity

The antioxidant activity of the three extracts (*n*-hexane, CH_2_Cl_2_ and EtOAc) was quantified using four assays: DPPH^•^ (2,2-diphenyl-1-picrylhydrazyl) and ABTS^·+^ (2,2′-azino-bis (3-ethylbenzothiazoline-6-sulfonic acid), which measure radical scavenging activity; and phenanthroline and FRAP (Ferric Reducing Antioxidant Power), which evaluate the reducing potential of antioxidants. Table 2 provides a summary of the obtained results.

Significant differences were observed among extracts (*p* ˂ 0.05). The EtOAc extract exhibited the strongest activity across all assays, with an IC_50_ of 15.68 ± 1.51 μg/mL in DPPH^•^, 24.29 ± 0.28 μg/mL in ABTS^·+^, and A_0.5_ of 19.97 ± 2.40 μg/mL in phenantroline, and 22.86 ± 1.37 μg/mL in FRAP. The CH_2_Cl_2_ extract showed a lower but still moderate activity, while the *n*-hexane extract exhibited the weakest activity. Although the standard ascorbic acid (3.07–4.40 μg/mL) and trolox (3.27–5.43 μg/mL) showed the highest antioxidant activity in all the cases, our findings reveal that *V. agnus-castus* leaf extracts, particularly the EtOAc extract, demonstrate stronger antioxidant activity compared to common values reported in previous studies on plant extracts. For instance, Rashed [23] evaluated the hexane, dichloromethane, and EtOAc extracts from the aerial parts of *V. agnus castus* and observed the highest activity with the EtOAc extract, which achieved an 88% inhibition at 100 µg/mL in the DPPH^•^ free radical scavenging assay. Similarly, Kımna and Fafal [9] reported IC_50_ values ranging from 132 to 800 μg/mL for leaf and fruit extracts using the same DPPH^•^ assay, indicating a lower antioxidant capacity compared to our results. Additionally, Boujbiha et al. [24] investigated fruit extracts and obtained IC_50_ and A_0.5_ values between 160 and 3108 μg/mL across several assays, including DPPH^•^, ABTS^•+^, and FRAP, further highlighting the comparatively higher efficacy of the extracts evaluated in our study.

The antioxidant activity of the tested extracts strongly correlated with their chemical compositions, as revealed by NMR and GC-MS analysis. The extraction solvent played a critical role in determining the bioactive compounds present in each extract, which ultimately influenced their level of activity in the antioxidant assays. The EtOAc extract exhibited superior antioxidant activity across all four assays, a result directly attributable to its high content of phenolic compounds such as protocatechoic acid, caffeic acid, vanillic acid, and 4-hydroxybenzoic acid. These phenolic compounds are known for their potent antioxidant properties, primarily due to their ability to donate electrons or hydrogen atoms to neutralize free radicals and prevent oxidative damage [25,26]. Additionally, the presence of 4-coumaric acid, which has been reported to exhibit strong radical-scavenging activity [25], further underscores the effectiveness of the extract. The dichloromethane extract demonstrated moderate antioxidant activity, which can also be attributed to the presence of phenolic compounds such as vanillic acid, 4-hydroxybenzoic acid, and 4-coumaric acid, although in lower concentrations compared to the EtOAc extract. In addition, compounds such as methyl caffeate and loliolide were detected, both of which possess documented antioxidant activities [27,28]. However, the moderate performance of this extract in the assays suggests that the concentration, solubility, or bioavailability of these compounds might not be as high as in the EtOAc extract. In contrast, the *n*-hexane extract displayed the weakest antioxidant activity, consistent with its chemical composition predominantly containing non-polar compounds. While some components are known to have antioxidant properties, such as β-caryophyllene [29], phytol [30], spathulenol [31], α-terpinylacetate, and caryophyllene oxide [32], their activity is typically less pronounced than that of phenolic compounds. Additionally, fatty acids like palmitic acid and linolenic acid are present in the extract, but their contribution to antioxidant activity is relatively limited due to their weaker radical-scavenging abilities. The low solubility of highly polar antioxidant compounds in non-polar solvents like *n*-hexane further explains the poor activity of this extract in all assays.

### 2.4. Determination of Acetylcholinesterase (AChE) and Butyrylcholinesterase (BChE) Inhibition Activity

The inhibitory activities of the three extracts against AChE and BChE were evaluated, revealing significant differences in their effects on the two enzymes (*p* ˂ 0.05). The IC_50_ values for the inhibition of both enzymes are presented in Table 3.

The enzymatic test revealed that only the dichloromethane extract had inhibitory effect on AchE, with an IC_50_ value of 786.16 ± 3.15 μg/mL, which is considered weak in comparison to galantamine, which presented an IC_50_ value of 7.14 ± 0.88 μg/mL. The inhibitory effect of this extract on BchE was more relevant. In comparison to galantamine, their respective IC_50_ values were 133.54 ± 1.45 and 35.41 ± 0.52 μg/mL. In contrast, both *n*-hexane and EtOAc extracts were less effective for the BchE inhibition test, with values of 577.34 ± 3.59 and 798.83 ± 0.75 μg/mL, respectively. Upon comparison with previous studies, the results obtained in this study reveal both similarities and differences in the cholinesterase inhibitory activity of plant extracts. For instance, Mota et al. [33] evaluated aqueous and ethanolic extracts of several plants, and reported that the aqueous extract of *V. agnus-castus* exhibited strong AChE inhibitory activity, while the ethanolic extract showed no inhibitory effect. Similarly, Kavaz et al. [34] investigated the ethanolic extract of *V. agnus-castus* seeds and reported significant AChE inhibition with an IC_50_ value of 36.34 ± 5.6 µg/mL. This is markedly more potent than the dichloromethane extract in the current study. The discrepancy might result from differences in the extraction solvent, plant part used, or extraction methods, or the specific bioactive compounds present in the extracts. Indeed, the low cholinesterase inhibitory activity observed for the *n*-hexane extract may be explained by its low content of phenolic compounds. Moreover, the inhibitory effect obtained with the dichloromethane extract is likely attributed to the presence of phenolic compounds such as 4-hydroxybenzoic acid, 4-coumaric acid [35,36], vanillic acid [36], and methyl caffeate [37], all of which have well-documented cholinesterase inhibitory properties. Additionally, loliolide, identified in the dichloromethane extract, has also been reported for its cholinesterase inhibitory activity [38], could also contribute to this activity. Despite its richness in phenolic compounds, including 4-hydroxybenzoic acid, vanillic acid, protocatechoic acid, 4-coumaric acid, and caffeic acid, the EtOAc extract demonstrated weak inhibition of BChE. This could be due to antagonistic effects between the active compounds. A previous study comparing the cholinesterase inhibitory activities of phenolic compounds, tested alone, in pairs, or in combination with several phenolic compounds or flavonoids, revealed that mixtures often exhibited lower inhibitory potency than individual compounds due to antagonistic interactions [39]. Therefore, to confirm these hypotheses and better understand the mechanisms underlying the observed inhibitory activities, future studies should focus on isolating and testing individual compounds from these extracts [40].

### 2.5. Antibacterial Activity

The results (Table 4) demonstrated that the three extracts from *V. agnus-castus* leaves exhibited inhibitory effects on the growth of three bacterial strains: *Staphylococcus aureus*, *Bacillus subtilis*, and *Escherichia coli*. The inhibition zone diameters varied, showing significant antimicrobial activity that was comparable, in some cases, to the standard antibiotic gentamicin. However, the extracts displayed no activity against *Pseudomonas aeruginosa* and *Salmonella typhimurium*, suggesting a selective antibacterial effect.

The most significant antibacterial effect was observed for the *n*-hexane extract, particularly against *S. aureus*, with an inhibition zone diameter of 22.33 ± 0.47 mm, and *Bacillus subtilis*, with a diameter of 18.33 ± 0.47 mm. This was followed by the dichloromethane extract, which showed activity against the same strains with inhibition zones of 18.67 ± 0.47 mm and 17.00 ± 0.00 mm, respectively. The EtOAc extract exhibited moderate activity, with inhibition zones ranging from 7.00 ± 0.00 mm to 10.33 ± 0.47 mm. The three extracts showed a rather weak activity against *E. coli*, with inhibition zones diameters varying from 7.00 ± 0.00 to 9.67 ± 0.47 mm, indicating a relative resistance of this strain or a lower affinity of the active compounds, which is consistent with the natural resistance of this Gram-negative strain to many antibacterial compounds [41]. Several studies, like that reported by Arokiyaraj et al. [42], have demonstrated the antibacterial potential of this plant, finding that leaf extracts could inhibit the growth of multiple resistant strains. Similarly, Balpinar et al. [43] reported that flower extracts showed inhibitory effects against mastitis pathogens. Berrani et al. [10] investigated methanolic extracts from various parts of *V. agnus-castus* (leaves, stems, flowers, roots, and seeds) and highlighted notable antibacterial activity, albeit with some variation depending on the plant part and bacterial strain tested. Boujbiha et al. [24] also reported moderate antibacterial effects of fruit extracts against all tested strains, further supporting the antimicrobial potential of this plant.

The antibacterial activity of the extracts was closely correlated with their chemical composition identified by NMR and GC-MS analyses. The *n*-hexane extract, showing a potent antibacterial activity especially against *S. aureus*, could be attributed to a high content in terpenoids like β-caryophyllene [44], spathulenol [45], caryophyllene oxide [46], phytol [47], and α-terpinyl acetate [48]. Additionally, fatty acids such as linolenic acid and palmitic acid have been reported to exhibit antibacterial activity [49,50]. These compounds are known for their antimicrobial properties, potentially resulting from their interactions with bacterial cell structures. The notable antibacterial activity of the dichloromethane extract can be linked to the presence of phenolic compounds, including vanillic acid, 4-hydroxybenzoic acid [51], 4-coumaric acid [52], and methyl caffeate [53], which are known for their ability to disrupt bacterial cell membranes. In addition, loliolide, a bioactive molecule, has been proven to exhibit antibacterial activity, and could also contribute to the potent activity of this extract [54]. Despite the richness in phenolic compounds of the EtOAc extract, such as 4-hydroxybenzoic acid, protocatechuic acid, caffeic acid, vanillic acid, and 4-coumaric acid, and in non-phenolic compounds like palmitic acid and tuberonic acid, known for their antibacterial activity, this extract displayed the weakest antibacterial activity. This lower efficacy may be attributed to insufficient concentrations of active compounds or the presence of antagonistic interactions between its constituents.

### 2.6. Molecular Docking Analysis

In the present study, DNA gyrase was selected as a target for molecular docking analysis due to its crucial role in bacterial DNA replication and its well-established involvement in the antibacterial activity of many plant-derived compounds. DNA gyrase is an essential enzyme in bacteria that introduces negative supercoils into DNA, a process necessary for DNA replication, transcription, and chromosome segregation [55]. Inhibiting this enzyme can lead to the accumulation of DNA damage, disrupting bacterial growth and viability [56]. Previous research has shown that DNA gyrase is a common target for antibiotics such as fluoro-quinolones, which exert their antibacterial effects by inhibiting the enzyme’s activity [57]. Given the antibacterial properties of *V. agnus-castus* extracts observed in our study, we hypothesized that the active compounds within these extracts may interact with DNA gyrase, potentially contributing to the observed antimicrobial effects. Therefore, docking analysis on DNA gyrase was conducted to further explore the possible mechanisms of action and to provide insights into the molecular interactions between the plant’s bioactive compounds and the enzyme, ultimately helping to identify promising candidates for further antimicrobial drug development.

The docking results for the reference molecule and the best-docked compounds for each studied enzyme are presented in Table 5. These results include binding energy values (in kcal/mol) and interactions, both hydrogen and hydrophobic, between the ligands and the target receptors.

**Figure 3:**
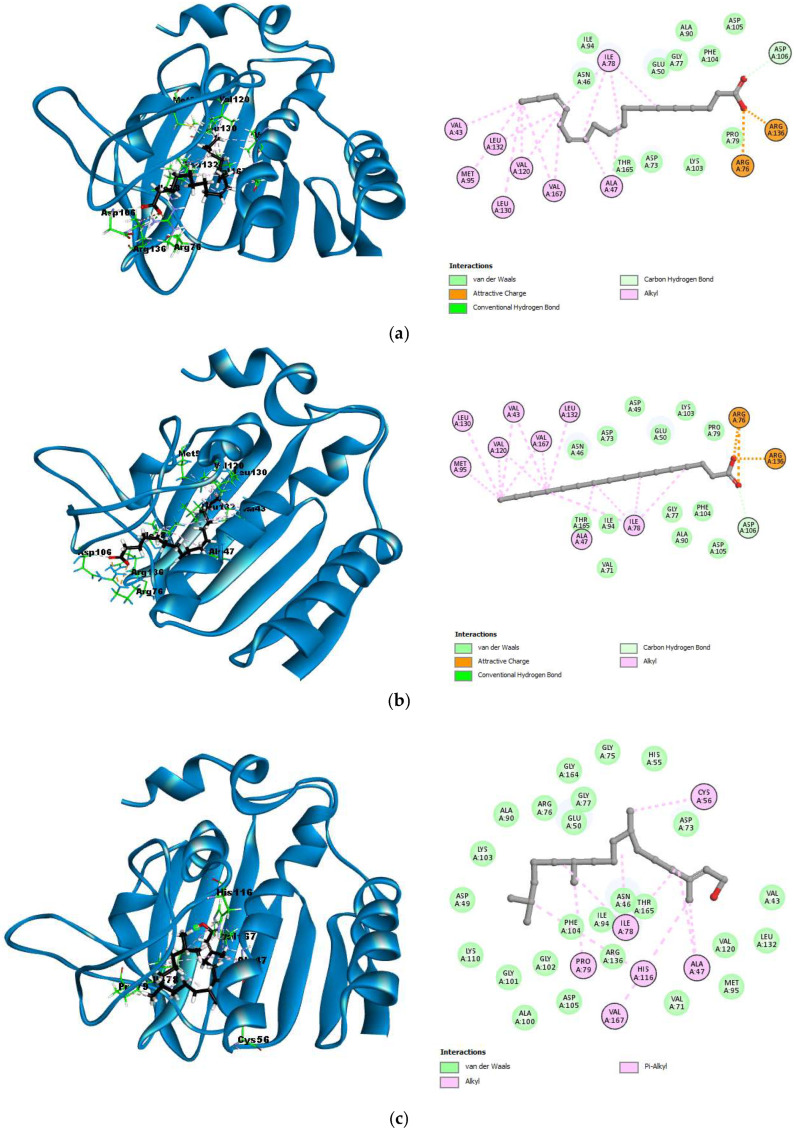
3D and 2D interactions between the best-docked compounds and DNA gyrase of *E. coli* (3G7E). (**a**) 3G7E–Linolenic acid; (**b**) 3G7E–Palmitic acid; (**c**) 3G7E–Phytol.

For the enzyme 3G7E (DNA gyrase of *E. coli*), the co-crystallized ligand (B46) exhibited a binding energy of −10.7579 kcal/mol and formed several hydrogen interactions with amino acids, such as Asp73, Asn56, Arg136, Gly102, Gly77, Arg76, and Asp49. Hydrophobic interactions were observed with Val43, Val120, Leu130, Val167, Met95, Ile94, His116, Ala47, Ile78, and Pro79. Among the best-docked compounds, linolenic acid had a binding energy of −7.6751 kcal/mol and interacted with Asp106 (2.45 Å) through hydrogen bonding, while its hydrophobic interactions included Val43, Leu132, Met95, Leu130, Val167, Ala47, and Ile78. Palmitic acid showed a binding energy of −8.0583 kcal/mol, with hydrogen bonds to Asp106 (2.89 Å), and hydrophobic interactions with Leu130, Met95, Val120, Val167, Leu132, Ala47, and Ile78. Phytol, with a binding energy of −8.5935 kcal/mol, did not form significant hydrogen bonds but showed hydrophobic interactions with Cys56, Ile78, Pro79, Val167, His116, and Ala47. These interactions are represented in Figure 3.

**Figure 4:**
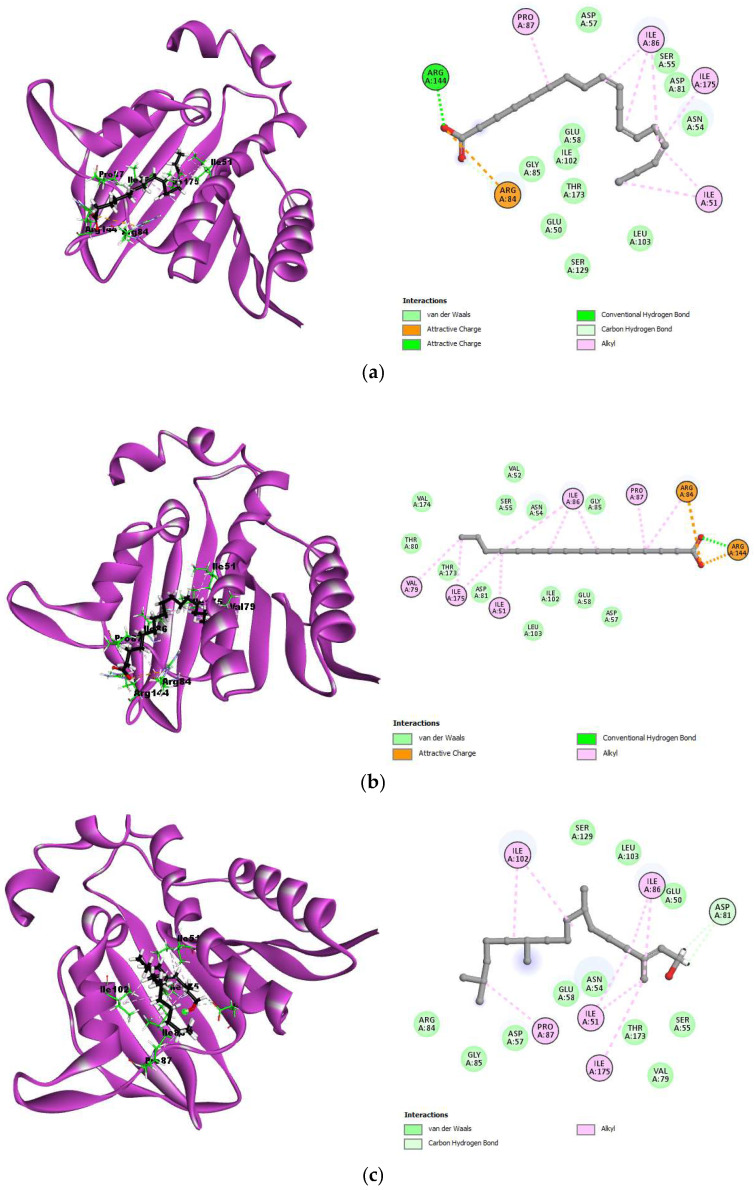
3D and 2D interactions between the best-docked compounds and DNA gyrase of *S. aureus* (3G75). (**a**) 3G7E–Linolenic acid; (**b**) 3G7E–Palmitic acid; (**c**) 3G7E–Phytol.

For 3G75 (DNA gyrase of *S. aureus*), the co-crystallized ligand (B48) had a binding energy of −6.1928 kcal/mol and formed hydrogen bonds with Asp81 (1.82 Å) and Ser55 (3.39 Å). Hydrophobic interactions were seen with Ile175, Val79, Ile51, Ile86, Gly85, and Asn54. Among the best-docked compounds, linolenic acid had a binding energy of −6.4867 kcal/mol and hydrogen-bonded with Arg144 (3.23 Å) and Arg84 (3.84 Å), while its hydrophobic interactions included Pro87, Ile86, Ile175, and Ile51. Palmitic acid showed a binding energy of −7.1954 kcal/mol, with hydrogen bonds to Arg144 (2.56 Å) and hydrophobic interactions with Val79, Ile175, Ile51, Ile86, and Pro87. Phytol exhibited a binding energy of −6.8489 kcal/mol, with hydrogen bonds to Asp81 (2.55 Å) and Asp81 (2.98 Å), and hydrophobic interactions with Ile102, Ile86, Ile51, Ile175, and Pro87. These interactions are depicted in Figure 4.

**Figure 5:**
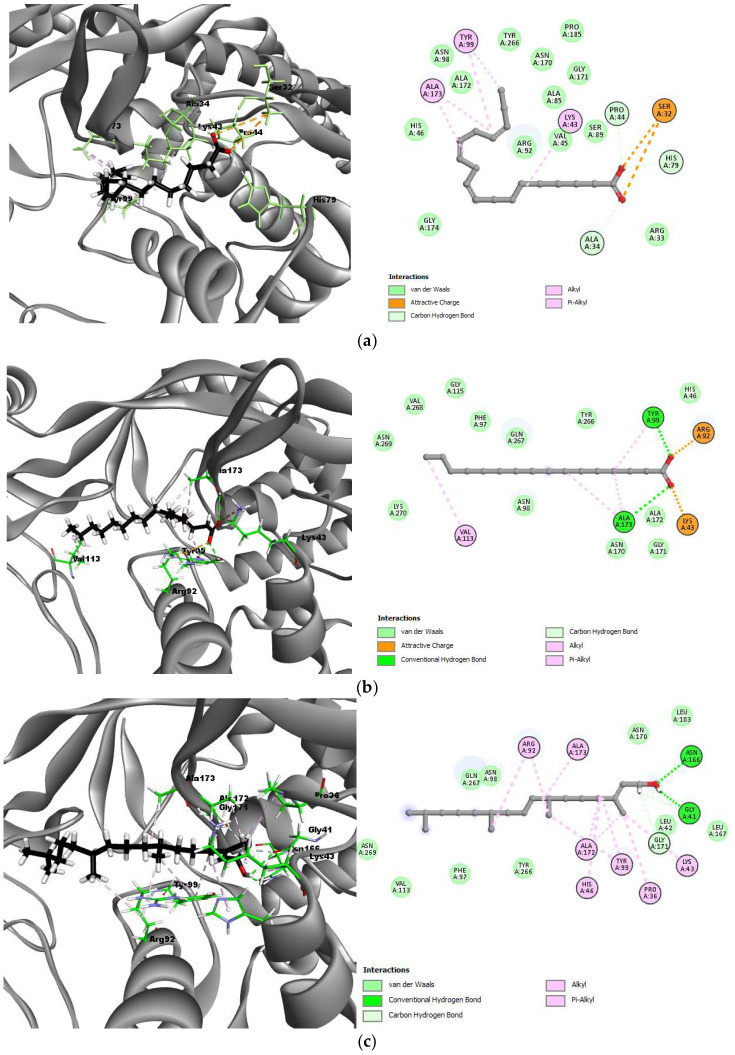
3D and 2D interactions between the best-docked compounds and DNA gyrase of *B. subtilis* (4DDQ). (**a**) 4DDQ–Linolenic acid; (**b**) 4DDQ–Palmitic acid; (**c**) 4DDQ–Phytol.

For 4DDQ (DNA gyrase of *B. subtilis*), the best-docked compounds showed binding energies of −6.3265 kcal/mol for linolenic acid, −6.3731 kcal/mol for palmitic acid, and −6.6323 kcal/mol for phytol. Linolenic acid formed hydrogen bonds with Pro44 (2.65 Å), Ala34 (3.21 Å), and His79 (2.84 Å), while hydrophobic interactions were observed with Tyr99, Ala173, and Lys43. Palmitic acid showed hydrogen bonding to Tyr99 (2.51 Å) and Ala173 (3.05 Å), with hydrophobic interactions with Val113, Tyr99, and Ala173. Phytol interacted through hydrogen bonds with Asn166 (2.84 Å), Gly41 (3.15 Å, 3.45 Å), and Gly171 (3.66 Å), and hydrophobic interactions with Arg92, Ala173, Ala172, His46, Tyr99, Pro36, and Lys43. These interactions are shown in Figure 5.

Our results revealed distinct interactions between the phytocompounds and the target enzymes, with variations in binding energy and the nature of the interactions.

For *E. coli* (3G7E), linolenic acid, palmitic acid, and phytol showed significant binding, with linolenic acid demonstrating the strongest affinity, followed by palmitic acid. The compounds interacted mainly through hydrophobic forces, with linolenic acid also forming hydrogen bonds with Asp106. In *S. aureus* (3G75), linolenic acid and palmitic acid also displayed favorable docking scores, forming interactions with Arg144 and Arg84, indicating their potential against this pathogen. For *B. subtilis* (4DDQ), all compounds exhibited good binding, with linolenic acid showing the best docking results, primarily through hydrophobic interactions with Tyr99 and Ala173.

Overall, the results indicate that the phytocompounds from *V. agnus-castus*, especially linolenic acid and palmitic acid, could serve as potential candidates for antibacterial agents targeting DNA gyrase enzymes in *E. coli*, *S. aureus*, and *B. subtilis*. The variations in binding energy and the types of interactions highlight the importance of specific molecular interactions in determining the efficacy of these compounds.

The *n*-hexane extract was identified as the most potent against all bacterial strains, and previous chemical analyses confirmed the presence of three compounds palmitic acid, phytol, and linolenic acid in this extract. The molecular docking results showed that these compounds displayed strong binding affinities to DNA gyrase enzymes, particularly linolenic acid and palmitic acid, which demonstrated the best docking scores. The favorable binding profiles observed in the docking simulations are consistent with the high antibacterial activity of the *n*-hexane extract.

This correlation suggests that palmitic acid, phytol, and linolenic acid might be key contributors to the antibacterial effects observed in the *n*-hexane extract. These compounds could potentially be further explored as lead candidates for the development of natural antibacterial agents, with an emphasis on their ability to target DNA gyrase enzymes in *S. aureus* and *E. coli*. This study highlights the importance of both in vitro and in silico approaches in identifying and validating bioactive compounds for therapeutic purposes.

## 3. Materials and Methods

### 3.1. Chemicals and Reagents

Na_2_SO_4_, Folin–Ciocalteu reagent (FCR), sodium carbonate, gallic acid, aluminum nitrate, potassium acetate, quercetin, aluminum chloride (AlCl_3_), sodium acetate (C_2_H_3_NaO_2_), DPPH, ABTS, ferric chloride (FeCl_3_), phosphate buffer, potassium ferricyanide solution (K_3_[Fe(CN)_6_]), trichloroacetic acid (TCA), trolox, ascorbic acid, acetylthiocholine iodide, butyrylthiocholine chloride, 5,5′-dithiobis(2-nitrobenzoic acid) (DTNB), and galantamine were purchased from Sigma-Aldrich (Sigma-Aldrich, St. Louis, MO, USA). All other chemicals and solvents were of analytical grade. Bacterial strains were obtained from the Pasteur Institute, Algiers, Algeria.

### 3.2. Collection and Identification of the Plant

*V. agnus-castus* leaves were collected from Jijel in the province of Rabta Beach, Algeria, in September 2023. Then, they were dried in the air in the absence of light for two weeks. and finely ground using a grinder (Figure S4). The species was identified by Dr. Mohamed Moussaoui (Researcher at Pharmaceutical Sciences Research Center, Constantine, Algeria).

### 3.3. Preparation of Extracts

*V. agnus-castus* leaf powder (20 g) was mixed with 300 mL of methanol (MeOH)/H_2_O (1:1). The suspension was homogenized and left for 24 h at room temperature. A Whatman N°1 paper was used to filter the suspension, and the filtrate was concentrated under reduced pressure to get rid of the methanol. The aqueous phase was extracted with *n*-hexane (3 × 300 mL), then CH_2_Cl_2_ (3 × 300 mL), and finally with EtOAc (3 × 300 mL). The organic phases were separately combined and dried by adding Na_2_SO_4_, and evaporated at 40 °C using a rotavapor (Heidolph, Schwabach, Germany), giving three different residues [58].

### 3.4. Determination of TPC, TFC, and TFolC

#### 3.4.1. Determination of Total Phenolic Content (TPC)

The TPC of the three fractions was assessed using the Folin–Ciocalteu Reagent (FCR) method [59]. A 20 µL volume of each extract was mixed with 80 µL of sodium carbonate (7.5% *w*/*v*) and 100 µL of FCR (diluted 1:10 with deionized water). The reaction mixture was incubated in darkness for two hours. The absorbance of the resulting color was measured at 765 nm. Gallic acid served as the standard to generate a calibration curve (Y = 0.0041X + 0.207; R^2^ = 0.9738), which was used to calculate the TPC. The results were expressed as µg of gallic acid equivalent per mg of dry extract.

#### 3.4.2. Determination of Total Flavonoids Content (TFC)

With minor modifications, the aluminum nitrate colorimetric assay described in the literature [60] was applied to determine the TFC in the *n*-hexane, dichloromethane, and EtOAc extracts. A 50 µL volume of each fraction at various concentrations was combined with 10 µL of potassium acetate (CH_3_COOK; 9.8% *w*/*v*), and 10 µL of aluminum nitrate (Al(NO_3_)_3_·9H_2_O). The mixture was incubated for 40 min, and the absorbance was subsequently measured at 415 nm. To prepare the blank, methanol was used instead of the extracts. The TFC was determined using the quercetin calibration curve (Y = 0.0085X + 0.0471; R^2^ = 0.9954). The results were expressed as µg of quercetin equivalents per mg of dry extract.

#### 3.4.3. Determination of Total Flavonol Content (TFolC)

For this assay, a 50 µL volume of each extract at various dilutions was combined with 50 µL of AlCl_3_ (2%) and 150 µL of C_2_H_3_NaO_2_ (5%). The mixture was incubated in darkness for 90 min, followed by absorbance measurement at 440 nm. Methanol was used as the blank in place of the reagents. Quercetin was used to generate a calibration curve (Y = 0.0109X + 0.1081; R^2^ = 0.9979), and the TFolC was expressed as µg of quercetin equivalents per mg of dry extract [61].

### 3.5. GC-MS and NMR Analyses

GC-MS data were performed on the raw crude extracts directly and after trimethylsilylation with *N*,*O*-bis(trimethylsilyl)trifluoroacetamide (BSTFA) (Fluka, Buchs, Switzerland). GC-MS analyses were conducted with an Agilent 6850 GC (Milan, Italy), equipped with an HP-5MS capillary column (stationary phase: 5%–phenyl-methylpolysiloxane); length: 30 m; ID: 0.25 mm; film thickness: 0.25 µm), coupled to an Agilent 5973 Inert MS detector operated in the full scan mode (*m*/*z* 40–550) at a frequency of 3.9 Hz and with the EI ion source and quadrupole mass filter temperatures kept, respectively, at 200 °C and 250 °C. Helium was used as carrier gas at a flow rate of 1 mL/min. The injector temperature was 250 °C, and the temperature ramp raised the column temperature from 70 °C to 280 °C: 70 °C for 1 min; 10 °C/min until reaching 170 °C; and 30 °C for 1 min until reaching 280 °C. Then, it was held at 280 °C for 5 min. The solvent delay was 4 min. Metabolites were identified by comparing their EI mass spectra at 70 eV with mass spectra collected in the NIST 20 mass spectral library (available online: https://www.nist.gov/srd/nist-standard-reference-database-1a (accessed on 14 January 2025). Moreover, the identification was supported by the Kovats retention index (RI) calculated for each analyte by the Kovats equation, using the standard n-alkane mixture in the range C7–C40.

A Bruker 400 Anova Advance (Karlsruhe, Germany) spectrometer was used to record the proton nuclear magnetic resonance (^1^H NMR) at 400 MHz. Deuterated chloroform (CDCl_3_) and deuterated methanol (CD_3_OD) were used as solvents and internal standards.

### 3.6. Evaluation of Antioxidant Activity

The antioxidant activity of the three extracts was carried out on 96-well microplates by four different methods: DPPH free radical scavenging, ABTS cation radical assay, FRAP, and reduction by the formation of the Fe2^+^-phenanthroline complex. Trolox and ascorbic acid were used as standards.

(a) DPPH scavenging assay

For this test, a volume of 160 μL of DPPH (0.1 mM) was added to 40 μL of the samples diluted in methanol to different concentrations. After incubating for 15 min, the absorbance was measured at 517 nm [62]. The DPPH radical scavenging capacity was calculated using the following equation:

$$DPPH\;scavenging\;effect\left(\%\right)=\frac{A_{Control}-A_{sample}}{A_{Control}}\times 100$$

where

A_control_: negative control absorbance
A_sample_: extract (or standard) absorbance

(b) ABTS cation radical assay

The test was carried out by adding 160 μL of ABTS^•+^ solution to 40 μL of each extract (dissolved in methanol at different concentrations), and the absorbance was measured at 734 nm after 10 min of incubation in the dark. Methanol and ABTS^•+^ solution were used as the blank. The same DPPH formula was used to compute the inhibition percentage, as explained [63].

(c) Phenanthroline assay

For this test, a mixture of 10 μL of each extract (diluted at different concentrations), 50 μL of FeCl_3_ (0.2%), 30 μL of phenanthroline (0.5%), and 110 μL of methanol was prepared. The mixture was then incubated in the dark at 30 °C for 20 min, and the absorbance was measured at 510 nm [64].

(d) FRAP test

A volume of 10 μL of each extract at different concentrations was mixed with 40 μL of phosphate buffer (pH 6.6) and 50 μL of ferricyanide potassium solution (K_3_ [Fe (CN)_6_] (1%). After incubation at 50 °C for 20 min, the reaction was stopped by the addition of 50 μL of trichloroacetic acid (TCA) (10%). Subsequently, 40 μL of distilled water and 10 μL of anhydrous iron chloride solution (0.1%) were added. Finally, the absorbance was measured at 700 nm using methanol as a blank [65]. The results were expressed as IC_50_ values (μg/mL), corresponding to the 50% inhibition concentration, in both DPPH and ABTS tests, and as A_0.5_ (μg/mL), corresponding to the concentration indicating 0.50 of absorbance, in Phenanthroline and FRAP assays.

### 3.7. Determination of AChE-and BChE Inhibition Activity

The enzymes studied were AChE and BChE, with acetylthiocholine iodide and butyrylthiocholine chloride, respectively, as specific substrates. 5,5′-dithiobis(2-nitrobenzoic acid) (DTNB) was used as a dye reagent to detect enzymatic activities. The experimental protocol consisted of mixing 150 μL of sodium phosphate buffer (100 mM, pH = 8.0) with 10 μL of each extract dissolved in methanol, prepared at different concentrations. To this mixture, 20 μL of AChE solution (5.32 × 10^−3^ U) or BChE solution (6.85 × 10^−3^ U) was added, the mixture was incubated at 25 °C for 15 min. Subsequently, 10 μL of DTNB (0.5 mM) and 10 μL of the corresponding substrate, either acetylthiocholine iodide (0.71 mM) or butyrylthiocholine chloride (0.2 mM), was added. Measurements of absorbance were taken at 412 nm immediately (after adding the substrates), and then after 5, 10, and 15-min intervals. The results were expressed as IC_50_ values (μg/mL). For this assay, galantamine was selected as the positive control [66].

### 3.8. Evaluation of Antibacterial Activity

The antibacterial activity of the three extracts was sought by the well diffusion method against ATCC test bacteria (American Type Culture Collection), which are *Staphylococcus aureus* (ATCC 25923), *Bacillus subtilis* (ATCC 6633) *Escherichia coli* (ATCC 25922), *Pseudomonas aeruginosa* (ATCC 27853), and *Salmonella typhimurium* (ATCC 14028). The reactivation of the bacterial strains was carried out by seeding on selective media. Bacterial suspensions were prepared from the 18 h cultures. The cell density of each suspension was adjusted by dilution in sterile physiological water, and in comparison with the 0.5 McFarland solution [67]. The Petri dishes containing Muller–Hinton previously seeded with the bacterial strains were perforated to form wells of 6 mm in diameter. A volume of 40 μL of each extract (50 mg/mL in dimethylsulfoxide (DMSO)) was added to each well. The plates were left in a refrigerator for 30 min, and then incubated at 37 °C for 24 h. For this test, gentamicin served as the positive control, while DMSO was used as a negative control [68].

### 3.9. Statistical Analysis

Measurements for each treatment were conducted in triplicate. The data were statistically analyzed using SPSS software (version 25.0). Results were analyzed by one-way analysis of variance (ANOVA) followed by Tukey’s HSD post hoc test for multiple comparisons. Differences were considered significant at *p* < 0.05.

### 3.10. Molecular Docking Analysis

The molecular docking study was conducted to investigate the antibacterial activity of phytocompounds identified from *V. agnus-castus*. The chemical structures of the identified phytocompounds were obtained from PubChem and optimized for docking using the MOE (2015.10) software. The 3D coordinates of each compound were minimized to achieve the most stable conformation. The docking targets were selected based on the in vitro antibacterial results, which showed inhibition zones for *S. aureus*, *B. subtilis*, and *E. coli* exposed to the different extracts. The chosen target receptors included DNA gyrase structures from *S. aureus* (PDB ID: 3G75), *E. coli* (PDB ID: 3G7E), and *B. subtilis* (PDB ID: 4DDQ). These receptors were retrieved from the Protein Data Bank (PDB), and the structures were prepared by removing water molecules, adding hydrogen atoms, and optimizing them for docking. The docking simulations were then performed in MOE, where each extract was docked to the three target DNA gyrase structures to evaluate their potential antibacterial activity based on the binding affinity and interaction with the receptors [68].

## 4. Conclusions

This study investigated the chemical profile and biological activities of *V. agnus-castus* leaf extracts from Algeria using both in vitro and in silico approaches. The extraction, performed with solvents of different polarities, led to the identification of several bioactive compounds via NMR and GC-MS. The antioxidant activity of the dichloromethane and EtOAc extracts was notable, while the dichloromethane extract showed moderate inhibition of BChE. The *n*-hexane extract demonstrated strong antibacterial activity, particularly against *S. aureus*, *B. subtilis*, and *E. coli.* Molecular docking simulations revealed that palmitic acid, phytol, and linolenic acid in this extract may be key contributors to its antibacterial effects. Further studies are required to validate their therapeutic potential.

## Figures and Tables

**Table 1:** TPC, TFC, and TFolC in extracts from *V. agnus-castus* leaves (*n* = 3).

Extract	TPC (µg GAE/mg)	TFC (µg QE/mg)	TFolC (µg QE/mg)
*n*-hexane	0.00 ± 0.00	0.00 ± 0.00	19.56 ± 0.51
Dichloromethane	86.82 ± 0.84	37.16 ± 0.50	21.39 ± 1.19
EtOAc	203.00 ± 1.86	47.36 ± 0.76	37.20 ± 1.80

**Table 2:** Antioxidant activity of *V. agnus-castus* extracts and reference compounds (ascorbic acid and trolox) evaluated via DPPH^•^, ABTS^·+^, phenantroline, and FRAP methods.

	IC_50_ (μg/mL)		A_0.5_ (μg/mL)	
	DPPH^•^	ABTS^·+^	Phenantroline	FRAP
***n*-Hexane**	366.58 ± 3.74 ^e^	>800	>200	>200
**CH_2_Cl_2_**	62.09 ± 2.23 ^d^	43.63 ± 0.58 ^c^	20.17 ± 1.65 ^c^	68.35 ± 2.14 ^d^
**EtOAc**	15.68 ± 1.51 ^c^	24.29 ± 0.28 ^b^	19.97 ± 2.40 ^c^	22.86 ± 1.37 ^c^
**Ascorbic acid**	4.40 ± 0.15 ^b^	3.07 ± 0.06 ^a^	3.08 ± 0.07 ^a^	3.76 ± 0.33 ^a^
**Trolox**	5.14 ± 0.12 ^a^	3.27 ± 0.24 ^a^	5.21 ± 0.09 ^b^	5.43 ± 0.44 ^b^

Values with different letters are significantly different at *p* < 0.05 according to Tukey’s HSD test.

**Table 3:** Results of AChE and BchE inhibition activity of *V. agnus-castus* extracts and the reference compound galantamine.

	IC_50_ (μg/mL)	
	BchE	AchE
***n*-Hexane**	577.34 ± 3.59 ^b^	-
**CH_2_Cl_2_**	133.54 ± 1.45 ^a^	786.16 ± 3.15 ^a^
**EtOAc**	798.83 ± 0.75 ^c^	-
**Galantamine**	35.41 ± 0.52 ^d^	7.14 ± 0.88 ^b^

-: not determined. Values with different letters are significantly different at *p* < 0.05 according to Tukey’s HSD test.

**Table 4:** Antibacterial activity of *V. agnus-castus* extracts and the antibiotic reference gentamicin.

Inhibition Zone (mm)					
	*S. aureus*	*B. subtilis*	*E. coli*	*P. aeruginosa*	*S. typhimurium*
***n*-Hexane**	22.33 ± 0.47 ^a^	18.33 ± 0.47 ^b^	9.67 ± 0.47 ^c^	-	-
**CH_2_Cl_2_**	18.67 ± 0.47 ^b^	17.00 ± 0.00 ^c^	7.00 ± 0.00 ^e^	-	-
**EtOAc**	10.33 ± 0.47 ^c^	9.00 ± 0.00 ^e^	7.00 ± 0.00 ^e^	-	-
**Gentamicin**	18.5 ± 0.5 ^b^	19.5 ± 0.71 ^b^	16.33 ± 0.47 ^c^	17.17 ± 0.25 ^c^	15.67 ± 0.70 ^d^

-: not determined. Values with different letters are significantly different at *p* < 0.05 according to Tukey’s HSD test.

**Table 5:** Docking results of the reference molecule and best-docked compounds for each studied DNA gyrase enzyme (3G7E, 3G75, and 4DDQ).

			Binding Energy (kcal/mol)	Hydrogen Interactions (Distance Å)	Hydrophobic Interactions
3G7E	Co-crystallized ligand	B46	−10.7579	Asp73 (1.69), Asn56 (1.88), Arg136 (2.46), Gly102 (2.82), Gly77 (2.60), Arg76 (3.05), Asp49 (2.52)	Val43, Val120, Leu130, Val167, Met95, Ile94, His116, Ala47, Ile78, Pro79
	Best-docked compounds	Linolenic acid	−7.6751	Asp106 (2.45)	Val43, Leu132, Met95, Leu130, Val167, Ala47, Ile78
		Palmitic acid	−8.0583	Asp106 (2.89)	Leu130, Met95, Val120, Val167, Leu132, Ala47, Ile78
		Phytol	−8.5935	-	Cys56, Ile78, Pro79, Val167, His116, Ala47
3G75	Co-crystallized ligand	B48	−6.1928	Asp81 (1.82), Ser55 (3.39)	Ile175, Val79, Ile51, Ile86, Gly85, Asn54
	Best-docked compounds	Linolenic acid	−6.4867	Arg144 (3.23), Arg84 (3.84)	Pro87, Ile86, Ile175, Ile51
		Palmitic acid	−7.1954	Arg144 (2.56)	Val79, Ile175, Ile51, Ile86, Pro87
		Phytol	−6.8489	Asp 81 (2.55), Asp 81(2.98)	Ile102, Ile86, Ile51, Ile175, Pro87
4DDQ	Best-docked compounds	Linolenic acid	−6.3265	Pro44 (2.65), Ala34 (3.21), His79 (2.84)	Tyr99, Ala173, Lys43
		Palmitic acid	−6.3731	Tyr99 (2.51), Ala173 (3.05)	Val113, Tyr99, Ala173
		Phytol	−6.6323	Asn166 (2.84), Gly41 (3.15), Gly41 (3.45), Gly171 (3.66)	Arg92, Ala173, Ala172, His46, Tyr99, Pro36, Lys43

## Data Availability

Data are contained within the article and Supplementary Materials.

## Supplementary Materials

The following supporting information can be downloaded at: https://www.mdpi.com/article/10.3390/molecules30030749/s1, Figure S1. ^1^H NMR spectrum of *Vitex agnus-castus n*-hexane extract recorded at 400 MHz in CDCl_3_; Figure S2. ^1^H NMR spectrum of *Vitex agnus-castus* dichloromethane extract recorded at 400 MHz in CDCl_3_; Figure S3. ^1^H NMR spectrum of *Vitex agnus-castus* EtOAc extract recorded at 400 MHz in CD_3_OD; Figure S4. Dried *Vitex agnus-castus* leaves employed in this study.
