# Supplementary material for: Bioactive Potential and Chemical Composition of Vitex agnus-castus L. Leaf Extracts Collected in Algeria: A Combined In Vitro and In Silico Approach
Source: Molecules. 2025 Feb 6;30(3):749. doi: 10.3390/molecules30030749 (PMC11820499; doi:10.3390/molecules30030749)
Supplement: Supplementary file 1 [file molecules-30-00749-s001.zip › molecules-3464785-supplementary.pdf]

molecules

Supplementary Materials

Bioactive Potential and Chemical Composition of *Vitex agnus-castus* L. Leaf Extracts Collected in Algeria: A Combined In Vitro and In Silico Approach

Amina Bramki [1], Ghozlane Barboucha [1], Ouided Benslama [2], Anna Andolfi [3], Fatima Zohra Makhlouf [1], Maria Smati [1], Djamila Benouchenne [1], Mohamed Moussaoui [4], Chawki Bensouici [5], Alessio Cimmino [3], Jesús G. Zorrilla [3,6], Maria Michela Salvatore [7] and Marco Masi [3,*]

[1] Higher National School of Biotechnology Taoufik KHAZNADAR, Nouveau Pôle Universitaire Ali Mendjeli, BP. E66, 25100 Constantine, Algeria; a.bramki@ensbiotech.edu.dz (A.B.); g.barb@ensbiotech.edu.dz (G.B.); makhlouf_f.zohra@umc.edu.dz (F.Z.M.); m.smati@ensbiotech.edu.dz (M.S.); d.benouchenne@ensbiotech.edu.dz (D.B.)
[2] Laboratory of Natural Substances, Biomolecules, and Biotechnological Applications, Department of Natural and Life Sciences, Larbi Ben M'Hidi University, 04000 Oum El Bouaghi, Algeria; ouided.benslama@univ-oeb.dz
[3] Department of Chemical Sciences, University of Naples Federico II, 80126 Naples, Italy; andolfi@unina.it (A.A.); alessio.cimmino@unina.it (A.C.); jesus.zorrilla@uca.es (J.G.Z.)
[4] Pharmaceutical Sciences Research Center, 25100 Constantine, Algeria; mohamed.moussaoui@univ-bejaia.dz
[5] Biotechnology Research Center, UV 03, BP. E73, Ali Mendjeli, 25016 Constantine, Algeria; c.bensouici@crbt.dz
[6] Allelopathy Group, Department of Organic Chemistry, Facultad de Ciencias, Institute of Biomolecules, (IN-BIO), University of Cadiz, 11510 Puerto Real, Spain
[7] Department of Veterinary Medicine and Animal Production, University of Naples Federico II, 80137 Naples, Italy; mariamichela.salvatore@unina.it
* Correspondence: marco.masi@unina.it

Contents

Figure S1. ^{1}H NMR spectrum of *Vitex agnus-castus* *n*-hexane extract recorded at 400 MHz in $CDCl_3$.

Figure S2. ^{1}H NMR spectrum of *Vitex agnus-castus* dichloromethane extract recorded at 400 MHz in $CDCl_3$.

Figure S3. ^{1}H NMR spectrum of *Vitex agnus-castus* EtOAc extract recorded at 400 MHz in CD_3OD.

Figure S4. Dried *Vitex agnus-castus* leaves employed in this study.

molecules

MDPI

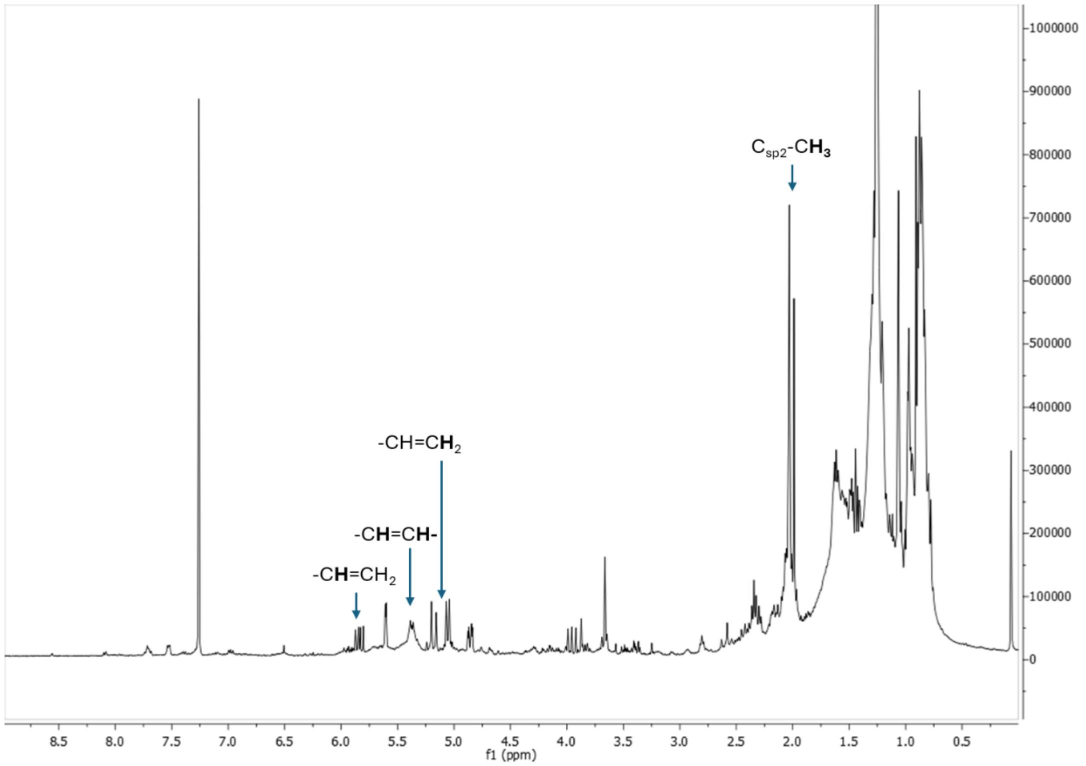

Figure S1. ^{1}H NMR spectrum of *Vitex agnus-castus n*-hexane extract recorded at 400 MHz in $CDCl_3$.

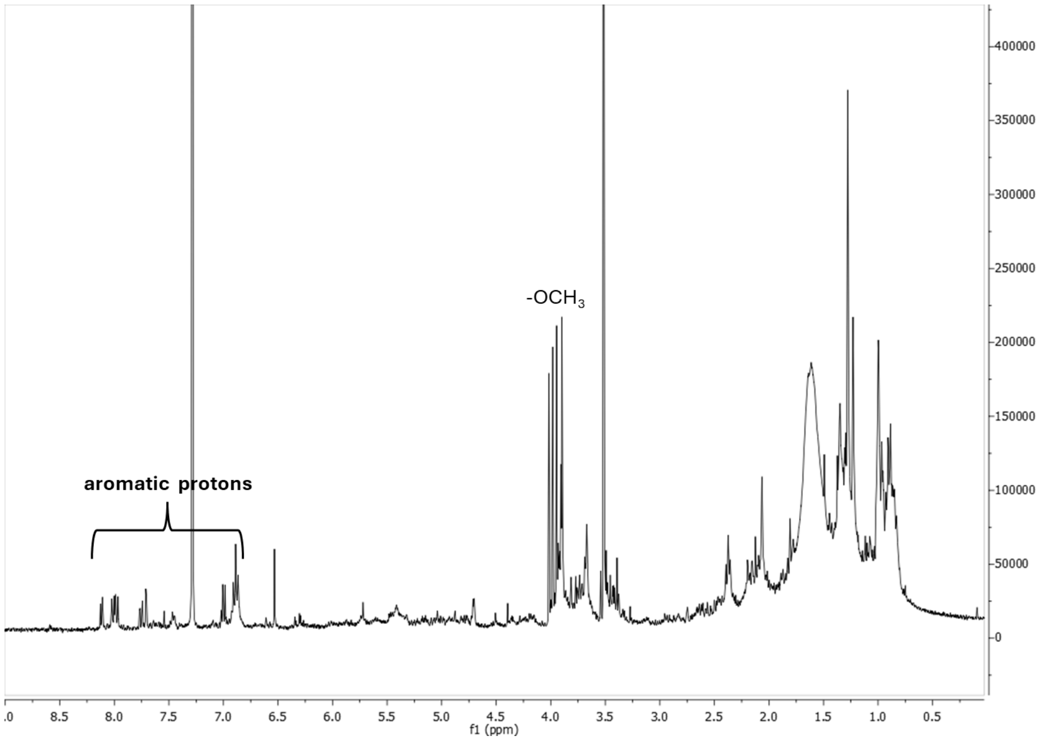

Figure S2. ^{1}H NMR spectrum of *Vitex agnus-castus* dichloromethane extract recorded at 400 MHz in $CDCl_3$.

molecules

MDPI

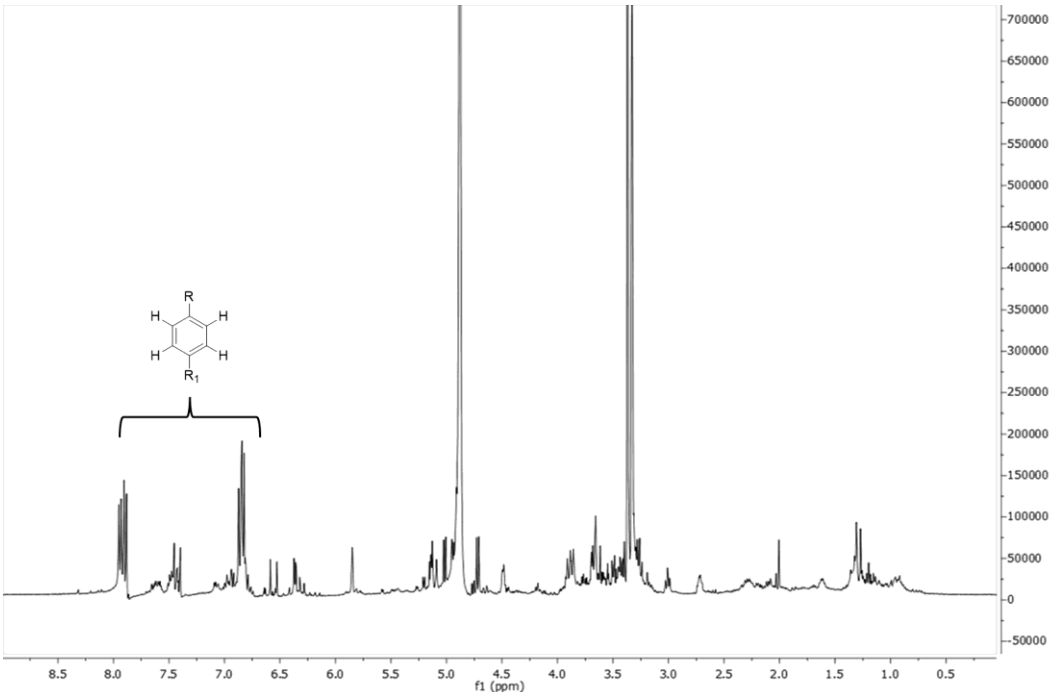

Figure S3. ^{1}H NMR spectrum of *Vitex agnus-castus* EtOAc extract recorded at 400 MHz in CD_3OD.

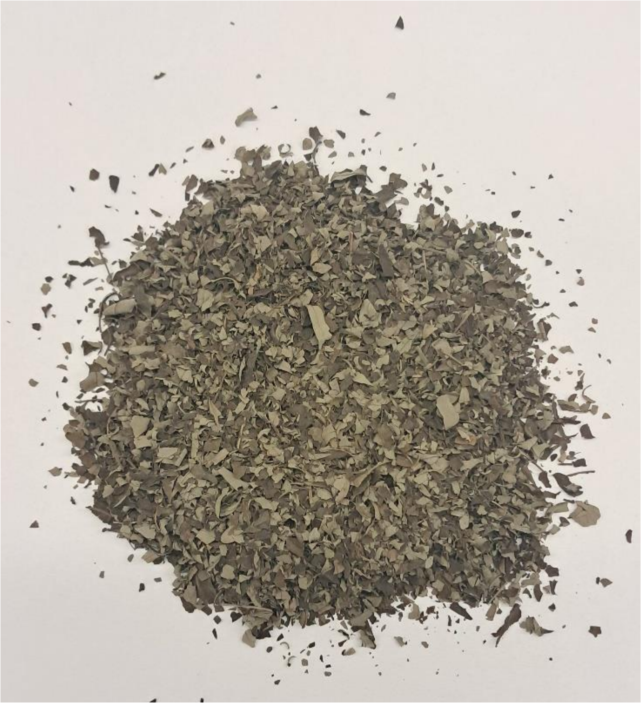

Figure S4. Dried *Vitex agnus-castus* leaves employed in this study.
